# Emphysematous Pyelonephritis Caused by a Giant Fecaloma

**DOI:** 10.1155/2019/8743525

**Published:** 2019-12-19

**Authors:** Mikael Abi Abdallah, Nehme Raad, Naim Yarak, Jean Paul Noujeim, Antoine Noujeim

**Affiliations:** ^1^Division of Urology, Faculty of Medical Sciences, Lebanese University, Beirut, Lebanon; ^2^Department of Urology, Lebanese Geitaoui Hospital, Beirut, Lebanon

## Abstract

Emphysematous pyelonephritis (EPN) is a gas-producing necrotizing bacterial infection that involves the renal parenchyma and perirenal tissue. It is a life-threatening condition that requires a high index of suspicion, an early diagnosis and an aggressive treatment. Rapid progression to septic shock may occur. We report, to the best of our knowledge, the first case of obstructive EPN caused by a giant fecaloma. The patient was successfully treated with percutaneous drainage and broad-spectrum antibiotics, in addition to fecaloma evacuation using fleet enemas and oral laxatives. This shows how fecal impaction, a common pathology in routine clinical practice, can cause some serious complications if left untreated, including extrinsic ureteral compression.

## 1. Introduction

Emphysematous pyelonephritis (EPN) is an uncommon acute gas-producing necrotizing bacterial infection that involves the renal parenchyma and its surrounding areas. It is a life-threatening condition that requires a high index of suspicion, especially in patients who do not respond to the routine management of pyelonephritis. Although it is most commonly seen in diabetic cases, it has also been reported in patients with urinary tract obstruction without diabetes mellitus (DM). EPN deserves special attention due to its considerable mortality rate [1, 2].

We present, to the best of our knowledge, the first case of obstructive EPN caused by a giant fecaloma in a patient known to have uncontrolled DM and a history of recurrent urinary tract infections.

## 2. Case Presentation

A 70-year-old male patient, nonsmoker, known to have hypertension, uncontrolled DM, Alzheimer's disease, benign prostatic hyperplasia and a history of recurrent urinary tract infections, presented to our emergency department with two days history of high grade fever and decreased level of consciousness. As reported by his family, he was complaining of abdominal distension and constipation since one week without any episode of vomiting. The patient has been recently treated for urinary tract infection with an unknown antibiotic that was intentionally stopped two days prior to his presentation to our department.

Upon arrival, vital signs showed a temperature of 39°C, heart rate of 98 beats per minute, blood pressure of 100/60 mmHg and a respiratory rate of 20 breaths per minute. The patient appeared obnubilated with a Glasgow Coma Scale (GCS) of 12. Physical examination was relevant for left-sided costovertebral angle tenderness, abdominal distension, and fecal impaction on digital rectal examination.

Initial laboratory tests were consistent with sepsis likely secondary to urinary tract infection with blood tests revealing a white blood cell count (WBC) count of 8,100/mm^3^ with 76% granulocytes, hemoglobin of 10.4 g/dl, platelet count of 248 000/mm^3^, creatinine level of 1.23 mg/dl, urea of 71 mg/dl, and CRP of 175. Urine analysis demonstrated 6 to 8 WBCs, 2 to 3 RBCs, positive nitrites, and numerous bacteria.

**Figure 1:**
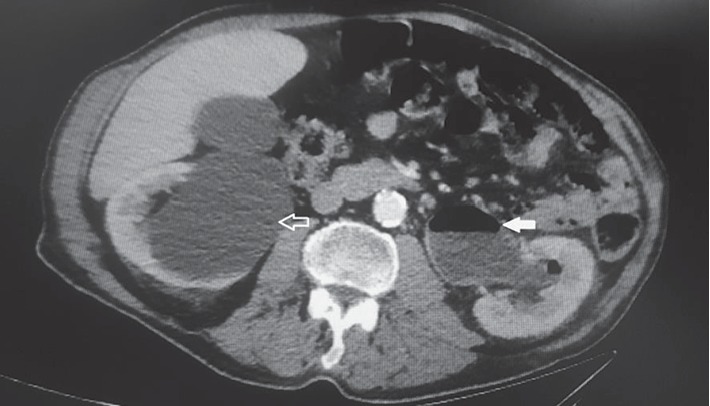
Computerized tomographic (CT) scan of the abdomen and pelvis. Left hydronephrosis with presence of air in the collecting system suggestive of left-sided emphysematous pyelonephritis (arrow). Right parapelvic cyst (empty arrow).

**Figure 2:**
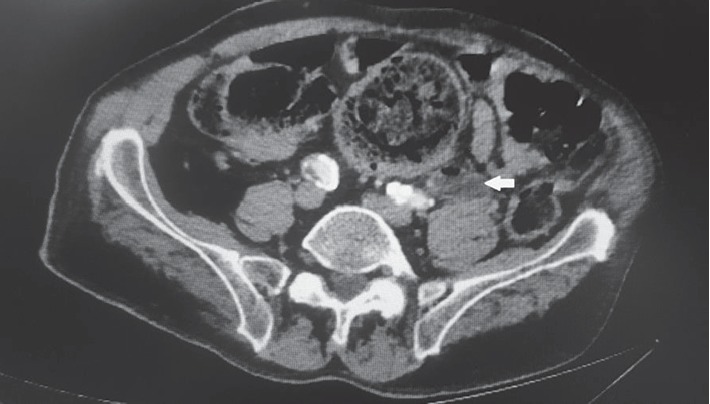
Computerized tomographic (CT) scan of the abdomen and pelvis. Left hydroureter with presence of intraluminal air (arrow).

**Figure 3:**
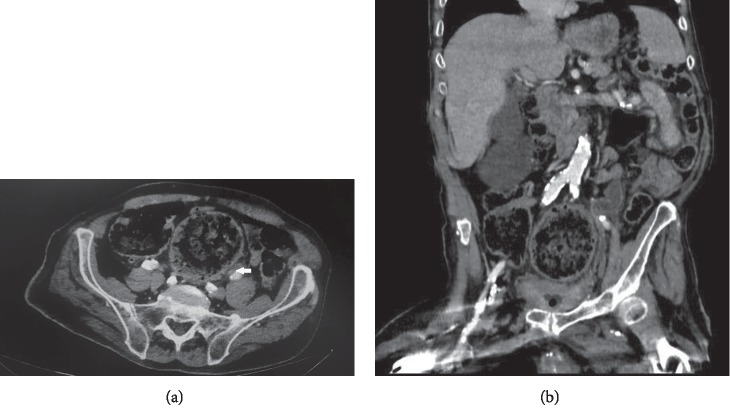
Computerized tomographic (CT) scan of the abdomen and pelvis ((a): axial view, (b): coronal view). Extrinsic compression of the left midureter (arrow) between an atherosclerotic external iliac artery and a giant fecaloma.

**Figure 4:**
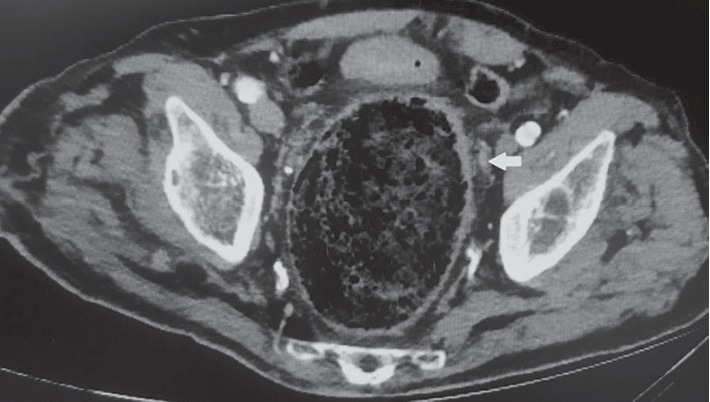
Computerized tomographic (CT) scan of the abdomen and pelvis. The left ureter (arrow) distal to the level of obstruction was not dilated.

Urgent CT scan of the abdomen and pelvis revealed severe left hydronephrosis and hydroureter with the presence of gas in the left collecting system and left ureter (Figures 1 and 2). The aforementioned hydroureteronephrosis resulted from an extrinsic compression of the left pelvic ureter between an atherosclerotic external iliac artery and a giant fecaloma (Figure 3). It is noteworthy that the ureter distal to the level of obstruction was not dilated (Figure 4).

Urine and blood cultures were taken. Initial medical management consisted of intravenous fluids, broad-spectrum antibiotics (Meropenem and a single shot of Amikacin) and strict glycemic control. After failure of initial conservative measures of fecaloma evacuation by digital rectal examination and fleet enemas, an ultrasound and fluoroscopy-guided left percutaneous nephrostomy of 10 French (Fr) was inserted for drainage of the renal pelvis. Frank pus was aspirated, confirming a left pyonephrosis. An antegrade ureterogram was intentionally omitted in order to avoid any retrograde bacteremia in this septic patient.

**Figure 5:**
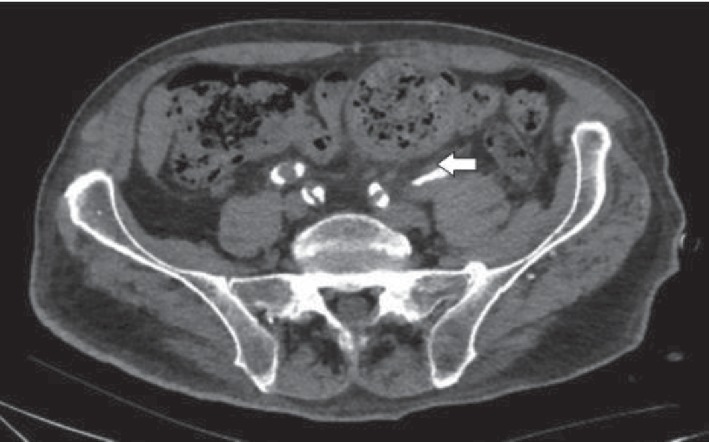
Follow-up noninjected computerized tomographic (CT) scan of the abdomen and pelvis. Resolution of the fecaloma and patency of the left ureter (arrow).

**Figure 6:**
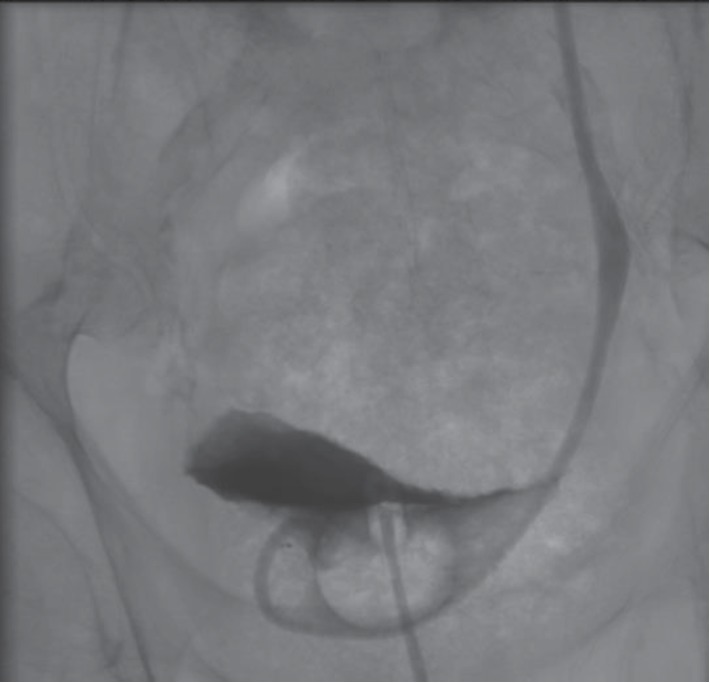
Antegrade ureterogram. Patency of the left ureter with absence of any intrinsic ureteral pathology.

A few hours following the nephrostomy insertion, the patient's confusion improved significantly. He became clinically and hemodynamically stable over the following days as the sepsis resolved. Oral laxatives and fleet enemas were given for fecaloma evacuation. Urine culture grew a multidrug-resistant Proteus Mirabilis. A follow-up noninjected CT scan was performed one week after the admission to document the resolution of the infection, the absence of renal abscesses and the resolution of the fecaloma (Figure 5). An antegrade ureterogram was performed showing a patent ureter with no intrinsic lesions (Figure 6); therefore the PCN was removed (in total nine days after patient's admission).

## 3. Discussion

Emphysematous pyelonephritis (EPN) is an uncommon acute gas-producing necrotizing bacterial infection that involves the renal parenchyma and perirenal tissue. It is a life-threatening condition that requires a high index of suspicion, especially in diabetic patients who do not respond to the routine management of pyelonephritis. Up to 90% of patients with EPN have uncontrolled DM. Other reported risk factors include drug abuse, end-stage renal disease, immunosuppression, neurogenic bladder, alcoholism, and anatomic anomalies. EPN is more common in females, and a urinary tract obstruction is present in 25–40% of cases [1–3].

Causative organisms include *Escherichia coli (most common), Proteus mirabilis, Klebsiella pneumoniae*, *Enterococcus* species, and *Pseudomonas aeruginosa* [1]. These micro-organisms are responsible for the production of gas (hydrogen and carbon dioxide) via the fermentation of glucose and lactate, especially in patients with high tissue glucose level, impaired tissue perfusion and immunodeficiency [3, 4].

The most common clinical features include fever/chills, flank pain, nausea, vomiting, and dysuria. These symptoms and signs are nonspecific and cannot differentiate EPN from other upper urinary tract infections [1]. Crepitus in the lumbar region, if present, can provide an important clinical clue to the diagnosis of EPN [4]. The left kidney was found to be more frequently involved than the right one [5].

Based on the radiological findings, multiple classifications for EPN were published, the most recent being published in 2000 by Huang and Tseng (Table 1). It showed a correlation between the class of EPN and its clinical outcome [3].

EPN requires an urgent attention. Rapid progression to septic shock may occur [3]. Thrombocytopenia, acute renal failure, hypotension (systolic blood pressure <90 mmHg), as well as disturbance of consciousness were found to be associated with a poorer prognosis and higher mortality [1, 2].

Once the diagnosis of EPN is made with a CT scan confirming the presence of intrarenal gas, an aggressive resuscitation should be given including oxygen, intravenous fluids, acid–base correction, combination of two broad-spectrum antibiotics, along with strict glycemic control. Inotropic support should be considered to maintain a systolic blood pressure >100 mmHg [3].

The treatment of EPN is controversial. Until the late 1980s, the accepted treatment of EPN has been emergency nephrectomy together with antibiotic therapy. However, surgery is often poorly tolerated in EPN due to poor hemodynamic status, resulting in a reported mortality rate of 40–50% [3]. Significant advances in imaging modalities, antibiotics, and image-guided drainage have shifted the management of EPN from invasive surgery to more conservative nephron-sparing approaches: medical management (MM), with percutaneous drainage (PCD) when indicated [3, 4]. Mortality rates have improved since the 1970s, and MM plus PCD was found to be associated with the lowest mortality rate of 13.5% [6].

**Figure 7:**
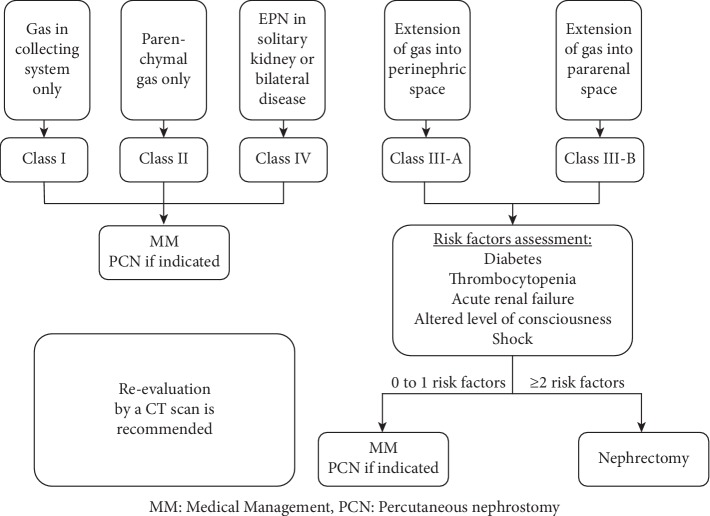
Management of EPN according to the classification of Huang and Tseng.

For localized EPN (classes 1 and 2), MM alone or combined with PCD provides generally a positive outcome. For extensive EPN (classes 3 and 4) with a benign course (0 or 1 risk factor), PCD combined with MM may be attempted first due to the high success rate (survival rate of 85%). PCD helps in the preservation of renal function in around 70% of cases. However, in patients with extensive EPN and a fulminant course (≥2 risk factors), MM plus PCD failed in 92% of cases and therefore nephrectomy should not be delayed in this setting (Figure 7). Patients who deteriorate or do not improve on conservative treatment require urgent nephrectomy (simple or radical/open or laparoscopic), with a reported mortality rate of 6.6% [2, 3].

A follow-up CT scan should be performed in patients on MM + PCD for EPN to evaluate the progression of the disease (formation of noncommunicating air-fluid collections) and the need for a more invasive treatment (nephrectomy) [7].

Our patient presented with a class I obstructive EPN (presence of gas in the collecting system only) caused by an uncontrolled DM and an extrinsic ureteral compression by a giant fecaloma. A conservative management (MM + PCD) was adopted as recommended with a favorable outcome.

## 4. Conclusion

Emphysematous pyelonephritis requires an early diagnosis and aggressive treatment. Mortality rates are high and rapid progression to septic shock may occur. Significant advances in image-guided drainage have shifted the management of EPN from invasive surgery to more nephron-sparing approaches. However, surgical intervention should not be delayed in patients with extensive disease or in those who do not improve on conservative treatment. This case report shows how fecal impaction, a common pathology in routine clinical practice, can cause some serious complications if left untreated, including extrinsic ureteral compression.

## Figures and Tables

**Table 1:** EPN classification by Huang and Tseng.

Class I	Gas in collecting system only
Class II	Parenchymal gas only
Class III-A	Extension of gas into perinephric space
Class III-B	Extension of gas into pararenal space
Class IV	EPN in solitary kidney or bilateral disease
